# Supplementary material for: Id1 and PD-1 Combined Blockade Impairs Tumor Growth and Survival of KRAS-mutant Lung Cancer by Stimulating PD-L1 Expression and Tumor Infiltrating CD8+ T Cells
Source: Cancers (Basel). 2020 Oct 28;12(11):3169. doi: 10.3390/cancers12113169 (PMC7693788; doi:10.3390/cancers12113169)
Supplement: Supplementary file 1 [file cancers-12-03169-s001.zip › cancers-957530-supplementary-1029/cancers-957530-supplementary xml-1028.docx]

**Figure S1.** Id1 inversely correlated with PD-L1 expression in patients from the TCGA LUAD data set stratified based on KRAS status. (**A**) KRAS-mutant TCGA LUAD cohort. (**B**) KRAS-wild type TCGA LUAD cohort.

**Figure S2.** Id1 promotes proliferation of KRAS-mutant murine LUAD cells. (**A**) Western blot for detection of Id1 protein in LLC, Lacun3, and 393P cells infected with shRNA lentiviral particles that target Id1, in comparison with the same cell lines infected with a control shRNA (pLKO-sc). (**B**) Cell proliferation assay (MTS) of 393P pLKO-sc and 393P Id1sh at day 0, 3, and 5 after plating. 393P cells with a reduced Id1 expression display significantly less proliferative potential than control cells (66.96 ± 3.98%, p < 0.001). (**C**) Cell proliferation assay (MTS) of Lacun3 pLKO-sc and Lacun3 Id1sh at day 0, 3, and 5 after plating. Lacun3 cells with a reduced Id1 expression showed a significant reduction in proliferative potential in comparison with Lacun3 pLKO-sc (35.49 ± 4.01%, p = 0.0004). (**D**) Cell-cycle analysis using an EdU incorporation assay of mutant and wild-type KRAS cell lines expressing a shRNA against Id1 or a pLKO-sc shRNA. Result is average of three independent experiments carried out in each cell line. The data are reported as the median with the interquartile range. * p < 0.05, *** p < 0.001, n.s. (not significant).

**Figure S3.** CD4^+^ T cells and NK cells have not anti-tumor functional effect in murine lung tumors after the *Id1* and PD-1 blockade. (**A**) Percentage of specific lysis in co-culture assay in LLC-GFP tumor cells (pLKO-sc or Id1sh) with OT-I CD8^+^ cells at different effector T cell:tumor cell ratios (pLKO-LLC TMG cells 60.63% [60.22–66.54] of specific lysis against Id1sh-LLC TMG cells 32.31% [29.46–33.10]; *p* = 0.0024). (**B**) In vivo tumor growth of *Id1*sh-LLC cells in C57BL/6J mice treated with NK^+^ cells depleting antibody and final tumor volumes at day 28 post-inoculation. (**C**) In vivo tumor growth of *Id1*sh-LLC cells in C57BL/6J mice treated with CD4^+^ T cells depleting antibodies and final tumor volumes at day 28 post-inoculation. Tumors were measured on days 7, 10, 14, 17, 21, and 24. The data are reported as the median with the interquartile range. ** p < 0.01, *** p < 0.001, n.s. (no significant).

**Figure S4.** The antitumor activity observed after the *Id1* and PD-1 blockade may be mediated by CD8^+^ T cells. (**A**). Flow-cytometric analysis of CD8^+^ effectors, naïve, effector memory, and central memory T cells in tumors harvested at day 14. (**B**) Flow-cytometric analysis of CD19^+^ T cells in tumors harvested at day 14. (**C**) Flow-cytometric analysis of NK1.1 cells in tumors harvested at day 14. (**D**) Flow-cytometric analysis of granulocytic and monocytic MDSCs cells in tumors harvested at day 14. (**E**) Flow-cytometric analysis of dendritic cells in tumors harvested at day 14. The data are reported as the median with the interquartile range. * p < 0.05, ** p < 0.01, n.s. (not significant).

**Figure S5.** Analysis of immune cell subpopulations in syngeneic tumors by flow cytometry. (**A**) Gating strategy for lymphoid cell (CD8 and CD4) populations. (**B**) Gating strategy for MDSCs.
