# Supplementary figures and images for: Id1 and PD-1 Combined Blockade Impairs Tumor Growth and Survival of KRAS-mutant Lung Cancer by Stimulating PD-L1 Expression and Tumor Infiltrating CD8+ T Cells
Source: Cancers (Basel). 2020 Oct 28;12(11):3169. doi: 10.3390/cancers12113169 (PMC7693788; doi:10.3390/cancers12113169)

Fig 1

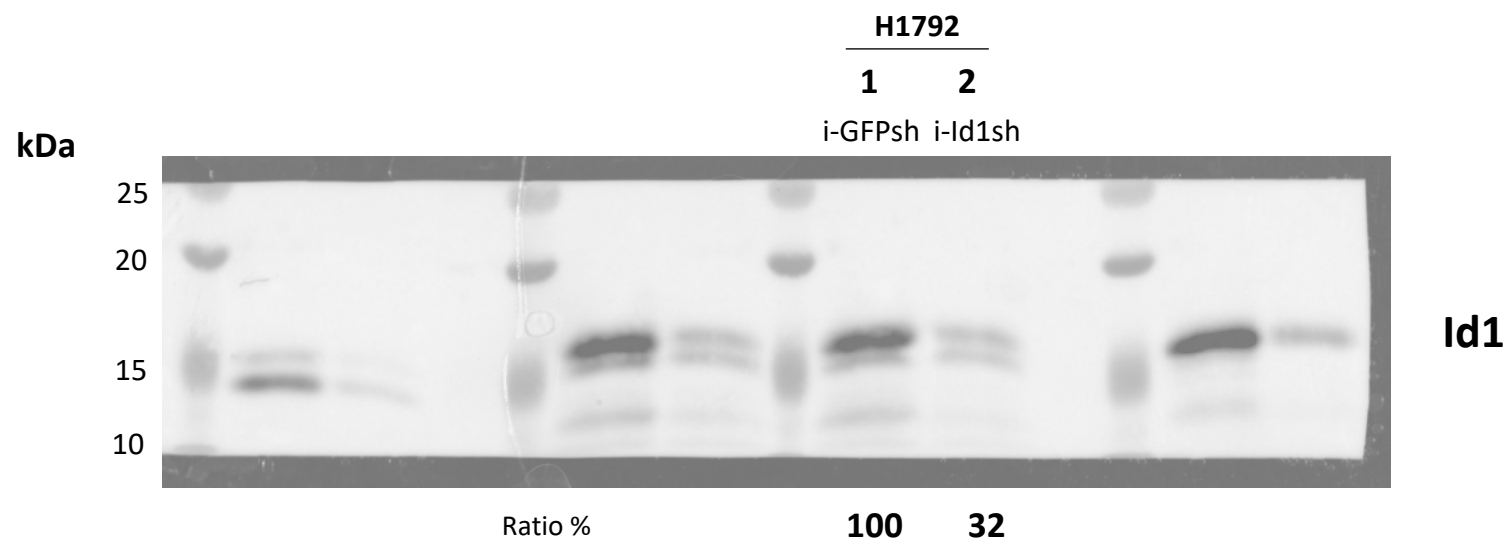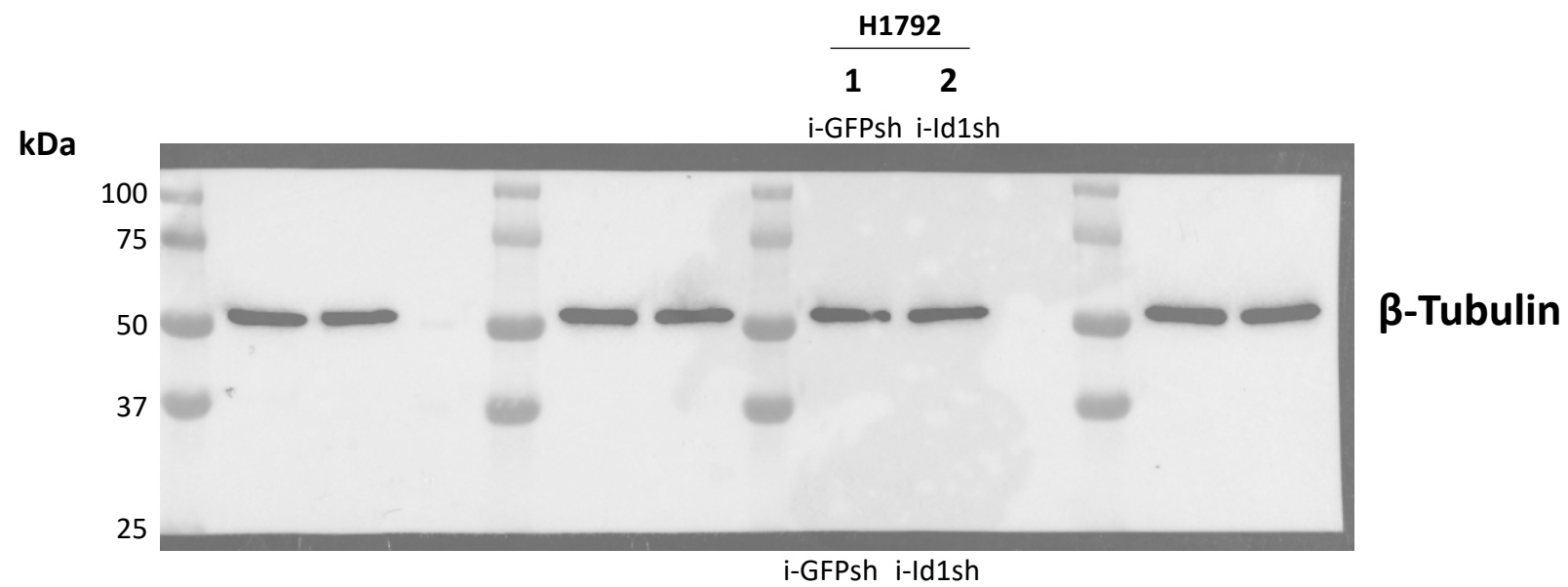

# Suppl Fig 3

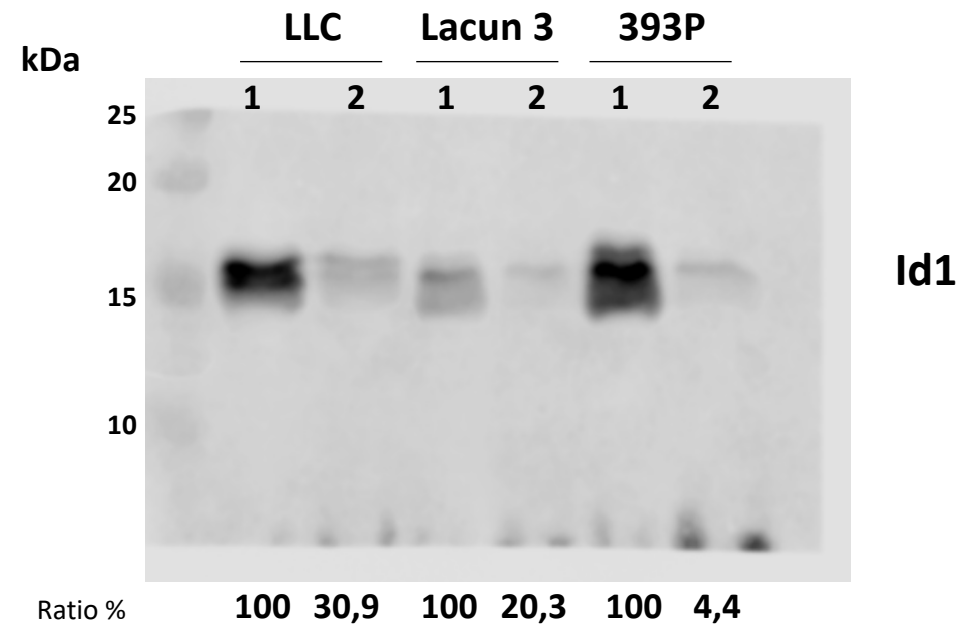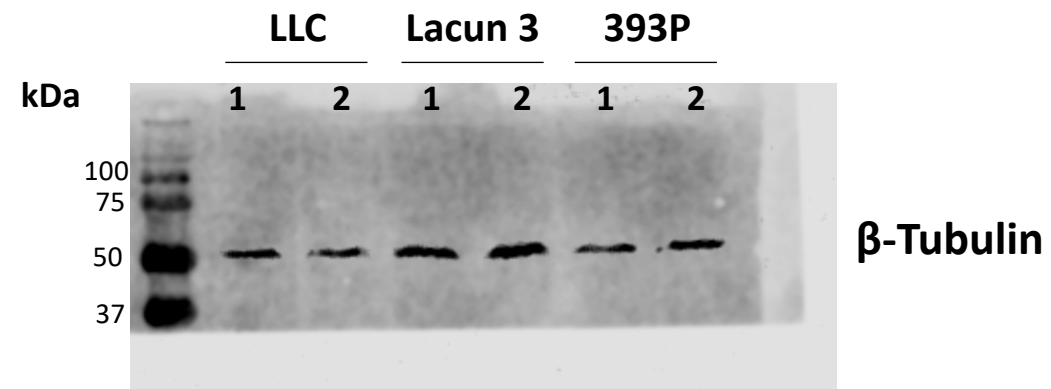

Supplement: Supplementary file 1 [file cancers-12-03169-s001.zip › cancers-957530-supplementary-1029/WB.pdf]
